# A Subdomain Interaction at the Base of the Lever Allosterically Tunes the Mechanochemical Mechanism of Myosin 5a

**DOI:** 10.1371/journal.pone.0062640

**Published:** 2013-05-01

**Authors:** Nikolett T. Nagy, Saikat Chakraborty, Gábor M. Harami, James R. Sellers, Takeshi Sakamoto, Mihály Kovács

**Affiliations:** 1 Department of Biochemistry, ELTE-MTA (Eötvös Loránd University-Hungarian Academy of Sciences) “Momentum” Motor Enzymology Research Group, Eötvös Loránd University, Budapest, Hungary; 2 Department of Physics and Astronomy, Wayne State University, Detroit, Michigan, United States of America; 3 Laboratory of Molecular Physiology, National Heart, Lung, and Blood Institute, Bethesda, Maryland, United States of America; Université de Genève, Switzerland

## Abstract

The motor domain of myosin is the core element performing mechanochemical energy transduction. This domain contains the actin and ATP binding sites and the base of the force-transducing lever. Coordinated subdomain movements within the motor are essential in linking the ATPase chemical cycle to translocation along actin filaments. A dynamic subdomain interface located at the base of the lever was previously shown to exert an allosteric influence on ATP hydrolysis in the non-processive myosin 2 motor. By solution kinetic, spectroscopic and ensemble and single-molecule motility experiments, we determined the role of a class-specific adaptation of this interface in the mechanochemical mechanism of myosin 5a, a processive intracellular transporter. We found that the introduction of a myosin 2-specific repulsive interaction into myosin 5a via the I67K mutation perturbs the strong-binding interaction of myosin 5a with actin, influences the mechanism of ATP binding and facilitates ATP hydrolysis. At the same time, the mutation abolishes the actin-induced activation of ADP release and, in turn, slows down processive motility, especially when myosin experiences mechanical drag exerted by the action of multiple motor molecules bound to the same actin filament. The results highlight that subtle structural adaptations of the common structural scaffold of the myosin motor enable specific allosteric tuning of motor activity shaped by widely differing physiological demands.

## Introduction

**Figure 1:**
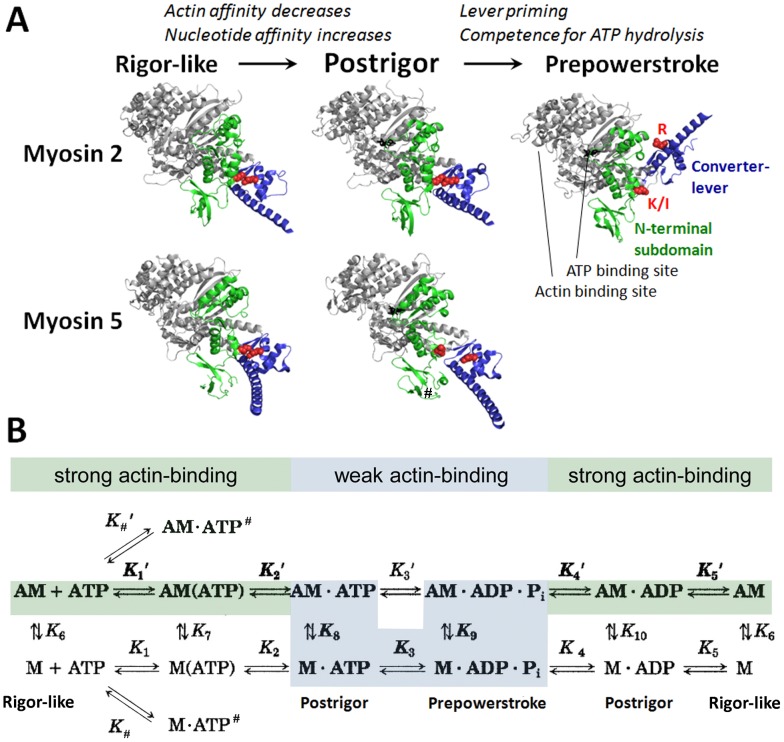
Structural and kinetic transitions of myosin. *A,* Nucleotide-induced structural changes of the myosin MD visualized by crystal structures of myosins 2 (upper row) and 5 (lower row) in the rigor-like, postrigor and prepowerstroke conformations (PDB codes: 3I5G, *Loligo pealei* (*Lp*) myosin 2 rigor-like; 3I5F, *Lp* myosin 2 postrigor; 1QVI, *Argopecten irradians* (*Ai*) myosin 2 prepowerstroke; 1OE9, *Gallus gallus* (*Gg*) m5a rigor-like; 1W7J, *Gg* m5a postrigor). The NTS (including the SH3-like domain) and the converter (including the proximal part of the lever) are shown in green and blue, respectively. Amino acid residues involved in the subdomain interaction investigated in this study are shown in red (K81 (*Lp* and *Ai* myosin 2) or I67 (*Gg* m5a) of the NTS, labeled ’K/I’; and R721 (*Lp* myosin 2) or R719 (*Ai* myosin 2) or R710 (*Gg* m5a) of the converter, labeled ’R’). Note that the separation of this residue pair occurs upon the postrigor-prepowerstroke transition in myosin 2, whereas it occurs upon the rigor-postrigor transition in m5a. Bound nucleotides are shown as black sticks. Light chains are omitted for clarity. See also **Table S1** for structural details. ***B,*** Kinetic scheme of the actomyosin mechanoenzymatic cycle, with the assignment of identified actin-detached myosin conformations. Upper and lower rows indicate enzymatic steps occurring in actin-attached (AM) and detached (M) forms of myosin, respectively. The main flux pathway is indicated by shading and bold characters. Strong and weak actin-binding states are shaded in green and cyan, respectively. In the main flux pathway, ATP binding to actomyosin occurs in two steps (a collision step (*K*_1_’) and a subsequent isomerization (*K*_2_’)). The myosin MD then rapidly dissociates from actin (*K*_8_). The *K*_3_ step involves the postrigor-prepowerstroke conformational transition and the chemical ATP hydrolysis step, which were not resolved kinetically in this study. Following ATP hydrolysis, M.ADP.P_i_ rebinds to actin (*K*_9_), and products are released (P_i_ in *K*_4_’ and ADP in *K*_5_’). Kinetic data (**Fig. 2, Fig. S1, Table S2**) indicate that I67K-m5aS1 and acto-I67K-m5aS1 can reversibly form an off-pathway ATP binding intermediate (M.ATP^#^ and AM.ATP^#^, respectively). Arrows for associating and dissociating species are omitted for clarity. All rate and equilibrium constants are defined in the rightward and downward directions. *K*_#_ and *K*_#_’ are defined as dissociation constants. A, actin; M, myosin; P_i_, phosphate.

Myosins are ubiquitous molecular motors of eukaryotic cells that utilize the free energy from ATP hydrolysis for unidirectional translocation along actin filaments [1]–[3]. The mechanochemical activity of different myosins drives a range of physiological processes ranging from muscle contraction, cell division and differentiation to membrane movements and intracellular transport. The motor domain (MD) of myosin, the core element driving mechanochemical action, contains the actin and ATP binding sites as well as the base of the myosin lever (**Fig. 1A**) [4]. The latter element amplifies structural changes occurring within the MD during the ATPase cycle to produce movement along actin. During the mechanochemical cycle, the binding of ATP to the actin-bound MD (step *K*_1_’ in **Fig. 1B**) weakens the actin affinity of different myosins by 3–6 orders of magnitude via allosteric changes (*K*_2_’) [5]–[10]. This process involves a structural transition from a strongly actin-bound nucleotide-free (rigor) to a strongly nucleotide-bound actin-detached (postrigor) state (**Fig. 1A**). The bound ATP then induces the priming of the myosin lever (a process termed recovery stroke, leading to the prepowerstroke conformation), which also brings catalytic residues into place for ATP hydrolysis (**Fig. 1A**; *K*_3_ in **Fig. 1B**) [11], [12]. After ATP is hydrolyzed (*K*_3_), the myosin.ADP.phosphate (P_i_) complex rebinds to actin (*K*_9_). This process triggers the kinetic activation of the release of hydrolysis products P_i_ and ADP (*K*_4_’ and *K*_5_’, respectively), linked to the strengthening of the actomyosin interaction, and the swing of the lever leading to the powerstroke [13], [14].

The MD is a complex structure comprising four subdomains, termed sequentially the N-terminal, upper and lower 50-kDa subdomains (NTS, U50 and L50, respectively) and the converter [4], [12]. Structural changes during the working cycle involve dynamic movements of subdomains. The NTS-converter interface is located at the base of the lever, remote from the actin and ATP binding sites (**Fig. 1A**). In the vast majority of myosin 2 isoforms, which are filament-forming motors driving muscle contraction and cell division, this interface harbors an unusual repulsive interaction between the side chains of a conserved lysine of the NTS and a conserved arginine of the converter (**Fig. 1A** **, Table S1**) [1]. Class 5 myosins, many of which are processive intracellular transporters, harbor a hydrophobic residue (mostly isoleucine) in the homologous position of the NTS (I67 in mouse myosin 5a (m5a)), whereas the converter arginine (R709 in mouse m5a) is conserved across many classes including myosins 2 and 5 [1]. In myosin 2 the repulsive interaction is maintained during the rigor-postrigor transition, whereas the interface is disrupted during lever priming (postrigor-prepowerstroke transition, **Fig. 1A**). In line with this, earlier we found that the removal or inversion of the positive charges of the NTS lysine and the converter arginine (via the K84M and R704E mutations, respectively, in *Dictyostelium discoideum* (*Dd*) myosin 2) specifically affected the lever priming step and, in turn, ATP hydrolysis and P_i_ release [15].

Available crystal structures of chicken m5a [16] indicate that the NTS-converter interface undergoes changes that are markedly different from those seen in myosin 2 (**Fig. 1A**). In the rigor-like state (crystallized in the absence of nucleotide and actin), the I67 and R710 (equivalent to R709 of mouse m5a) side chains are in close proximity, similar to the corresponding lysine and arginine residues of myosin 2 (**Fig. 1A** **, Table S1**). However, in contrast to myosin 2, these positions undergo a marked separation during the rigor-postrigor transition in m5a (**Fig. 1A** **, Table S1**). M5a has not been crystallized in the prepowerstroke conformation, but it is highly likely that in this state the NTS and the converter exhibit similar separation to that seen in the myosin 2 prepowerstroke state. The finding that the NTS-converter separation occurs during different steps of the mechanochemical cycles of myosins 2 and 5 implies that this interface may play a different role in the energy transduction mechanisms of myosins from different classes. To test this hypothesis, we introduced a positive charge, characteristic of myosin 2, into the NTS of mouse m5a via the I67K mutation. Our comprehensive biochemical, rapid transient kinetic and ensemble and single-molecule motility analysis of wild-type (wt) and I67K m5a constructs revealed that the NTS-converter interface indeed plays a specific and important role in the mechanochemical coupling mechanism of m5a. Most prominently, the mutational alteration of this interface perturbed the strong-binding actomyosin interaction and abolished the actin-induced activation of ADP release. These changes resulted in slower and mechanically sensitized processive motility of mutant m5a molecules.

## Results

### Proteins

To investigate the effect of the alteration of the NTS-converter interface on the mechanochemical mechanism of m5a, we introduced the I67K point mutation into single- and double-headed constructs of mouse m5a. The term “head” refers to the MD plus a neck (lever) region (an elongated α-helix with attached calmodulins). The single-headed subfragment-1 (S1)-like m5aS1 construct contained the MD and the first two IQ motifs of the neck with bound calmodulins, whereas the double-headed heavy meromyosin (HMM)-like m5aHMM construct contained an N-terminal GFP, the MD, all six IQ motifs with bound calmodulins and the proximal part of the coiled-coil tail. We used m5aS1 for solution kinetic and m5aHMM for motility experiments.

### I67K Mutant Retains Rapid ATP Binding and Hydrolysis but Forms an off-pathway Binding Intermediate

In line with earlier findings [9], [17], wt-m5aS1 showed a significant increase in steady-state tryptophan (Trp) fluorescence emission upon interacting with ATP or the non-hydrolyzable ATP analog adenylyl-imidodiphosphate (AMPPNP) (**Table 1**). This signal change was earlier assigned to the ATP-sensitive tryptophan located in the so-called relay loop (W483 in m5a), which reports the postrigor-prepowerstroke transition coupled to ATP hydrolysis (these two steps are embedded in *K*_3_ in **Fig. 1B**) [8], [18]–[20]. Also in line with earlier results [9], we found that ADP and adenosine 5′-O-(3-thio)triphosphate (ATPγS) failed to induce a similar increase in Trp fluorescence, resulting from the inability of these ligands to induce the prepowerstroke conformation to a detectable extent (**Table 1**). Upon interacting with various nucleotide ligands, I67K-m5aS1 showed similar Trp fluorescence changes to those of wt-m5aS1, suggesting that the mutant retained the capability of ATP binding and hydrolysis (**Table 1**).

**Table 1:** Functional properties of myosin 5a constructs^a^.

Parameter	Method of determination	wt	I67K
**Steady-state ATPase activity**	NADH assay		
Basal activity (s^−1^)^b^		0.020±0.03	0.015±0.04
Actin-activated *k*_cat_ (s^−1^)		9.9±0.8	1.8±0.4
* K*_actin_ (µM)^c^		5.2±0.3	1.0±0.4
* K*_ATP_ (µM)^d^		12±1	2.5±0.3
**Motility**			
Actin gliding (nm•s^−1^)^e^	Actin gliding assay	410±50	82±22
* v*_max_ (nm•s^−1^)	TIRF assay	410±30	250±40
* K*_ATP_ (µM)	TIRF assay	11±0.4	130±10
**Trp fluorescence change on nucleotide binding (%)**	Trp fluorescence		
ATP		17±3	9±1
AMPPNP		12±1	13±3
ATPγS		0±1	–1±1
ADP		–1±1	1±2
**ATP binding and hydrolysis**			
* K*_1_ *k*_2_ (µM^−1^s^−1^)^f^	Trp fluorescence	1.3±0.2	1.3±0.2
* k*_3_+ *k*_–3_ (s^−1^)^f^	Trp fluorescence	270±60	760±50
* k*_3_+ *k*_–3_ (s^−1^)^g^	Quenched-flow	>35	>39
* K*_3_	Quenched-flow	0.64±0.05	1.8±0.6
* K*_1_’*k*_2_’ (µM^−1^s^−1^)^f^	PA fluorescence, light scattering	0.73±0.08	1.2±0.2
* k*_2_’ (s^−1^)^f^	PA fluorescence, light scattering	>200	>300
**P_i_ release**	MDCC-PBP fluorescence		
* k*_4_ (s^−1^)^h^		0.029±0.003	0.025±0.002
* k*_4_’ (s^−1^)^i^		≥32	≥12
* A*_max_ (mol P_i_/mol m5aS1)^j^		0.99±0.01	0.90±0.02
**ADP interaction**			
* k*_5_ (s^−1^)	mdADP fluorescence	2.4±0.1	4.3±0.6; 1.3±0.1^k^
* k*_5_’ (s^−1^)	PA fluorescence	17±3	2.7±0.1
* K*_5_’ (µM)	PA fluorescence	3.7±0.8	1.0±0.3
**Actin interaction**	PA fluorescence		
* k*_–6_ (µM^−1^s^−1^)		28±1^l^	7.3±0.2
* k*_6_ (s^−1^)		1.1×10^−3l^	(8.1±0.8)×10^−3^
* K*_6_ (µM)		3.9×10^−5l^	1.1×10^−3^

a Solution kinetic data are for m5aS1 and motility data are for m5aHMM. Nomenclature of kinetic constants refers to **Fig. 1B**. All parameters were measured at 25°C. Mean ± SD values for two to four independent experiments are shown.

b At 1 mM ATP.

c Half-saturating actin concentration (at 1 mM ATP).

d Half-saturating ATP concentration (at 10 µM actin).

e At 1 mM ATP.

f Results of exponential analysis are shown (**Fig. 2**; rapid phase data for I67K-m5aS1). See also **Fig. S1** for global fitting results on I67K-m5aS1.

g Lower bounds set by maximal rate constant of rapid pre-steady state burst at 30 µM ATP.

h From steady-state slopes recorded at 100 µM ATP.

i Lower bounds set by maximal rate constant of rapid pre-steady state burst at 10 µM actin.

j Maximal amplitude of rapid pre-steady state burst at 10 µM actin.

k Rate constants of the rapid (40±3% amplitude) and slow phases of biphasic mdADP release.

l From ref. [17].

The ATP interaction kinetics of m5aS1 were further assessed in Trp fluorescence stopped-flow experiments. Upon rapidly mixing wt-m5aS1 with excess ATP in pseudo-first-order conditions, single-exponential transients were recorded with observed rate constant (*k*_obs_) values increasing hyperbolically with ATP concentration (**Fig. 2A–B**). As described for various myosins [8], [21], this behavior originates from a multistep ATP binding mechanism in which an initial collision step (*K*_1_ in **Fig. 1B**) is followed by structural transitions associated with changes in Trp fluorescence. In the case of m5aS1, the *K*_1_ and *K*_2_ steps cannot be resolved kinetically [8], [17]. Thus, the initial slope and the maximal *k*_obs_ of the fitted hyperbola will be determined by *K*_1_ *k*_2_ (the apparent second-order rate constant of ATP binding) and (*k*_3_+ *k*_–3_), respectively (**Table 1**) [8], [17].

**Figure 2:**
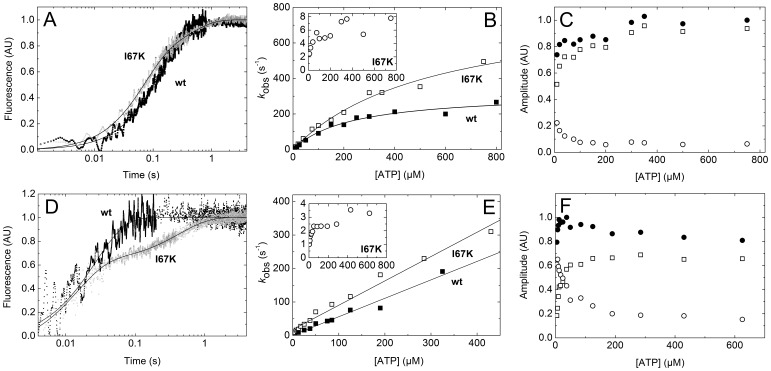
I67K mutation influences ATP binding to m5aS1 both in the absence and presence of actin. ***A,*** Normalized Trp fluorescence transients recorded on mixing 0.5 µM wt-m5aS1 with 7.5 µM ATP (black trace) or 0.5 µM I67K-m5aS1 with 10 µM ATP (gray trace) in the stopped-flow apparatus. Single exponential fit to the wt-m5aS1 trace shown yielded a *k*_obs_ of 7.1 s^−1^. I67K-m5aS1 traces were biphasic, with *k*_obs_ values of 15 s^−1^ (70% fractional amplitude) and 2.5 s^−1^ in the example shown. ***B,*** ATP concentration dependence of *k*_obs_ values obtained as in ***A*** (solid squares, wt-m5aS1; open squares and circles, I67K-m5aS1 rapid and slow phases, respectively). Hyperbolic fits to wt-m5aS1 and rapid-phase I67K-m5aS1 datasets shown in the main panel yielded maximal rate constants (*k*_3_+ *k*_–3_) of 320 s^−1^ and 820 s^−1^, respectively, with an initial slope (*K*_1_ *k*_2_) of 1.5 µM^−1^s^−1^ in both cases. ***C,*** ATP concentration dependence of rapid-phase (open squares), slow-phase (open circles) and total (solid circles) amplitudes of I67K-m5aS1 transients obtained as in ***A***. ***D,*** Normalized PA fluorescence transients recorded on mixing 0.35 µM PA plus 0.25 µM wt-m5aS1 (black trace) or I67K-m5aS1 (gray trace) with 50 µM ATP in the stopped-flow apparatus. Single exponential fit to the wt-m5aS1 trace shown yielded a *k*_obs_ of 37 s^−1^. I67K-m5aS1 traces were biphasic, with *k*_obs_ values of 71 s^−1^ (62% fractional amplitude) and 2.3 s^−1^ in the example shown. ***E,*** ATP concentration dependence of *k*_obs_ of transients obtained as in ***D*** (solid squares, wt-m5aS1; open squares and circles, I67K-m5aS1 rapid and slow phases, respectively). Linear fits to wt-m5aS1 and rapid-phase I67K-m5aS1 datasets shown in the main panel yielded slopes (*K*_1_’*k*_2_’) of 0.55 and 0.73 µM^−1^s^−1^, respectively. The *y* intercept was not significantly different from zero in wt-m5aS1, whereas it was 18 s^−1^ in I67K-m5aS1 in the example shown. ***F,*** ATP concentration dependence of rapid-phase (open squares), slow-phase (open circles) and total (solid circles) amplitudes of I67K-m5aS1 transients obtained as in ***D***. AU, arbitrary units.

In contrast to wt-m5aS1, biphasic Trp fluorescence transients were observed upon ATP binding to I67K-m5aS1 (**Fig. 2A–B**). The rapid phase *k*_obs_ values of the mutant were generally higher than those of wt-m5aS1, but showed similar ATP concentration dependence (**Fig. 2B** **,** **Table 1**). The slow phase *k*_obs_ values of I67K-m5aS1 increased moderately with ATP concentration (**Fig. 2B** inset). The amplitudes of the rapid and slow phases increased and decreased with ATP concentration, respectively, whereas the total amplitude showed early saturation indicating high overall ATP affinity (**Fig. 2C**). For reasons discussed in **Text S1**, we propose that the kinetic behavior of I67K-m5aS1 can be best explained by the formation of a reversibly ATP-bound off-pathway intermediate (M.ATP^#^ in **Fig. 1B**). We performed global fitting kinetic analysis to determine the kinetics of formation and dissociation of this intermediate (**Fig. S1A–B**, **Table S2**).

Upon ATP binding to acto-m5aS1, the initial collision step (*K*_1_’ in **Fig. 1B**) leads to a structural change (*K*_2_’) that weakens the actin affinity of m5aS1 by 5–6 orders of magnitude and leads to rapid dissociation of m5aS1 from actin (*K*_8_) [8], [9], [17]. We assessed these processes by monitoring changes in pyrene-labeled actin (PA) fluorescence and light scattering upon mixing acto-m5aS1 with excess ATP in the stopped-flow apparatus (**Fig. 2D**). In acto-wt-m5aS1, the *k*_obs_ values of the recorded single-exponential PA fluorescence transients increased quasi-linearly in the examined ATP concentration range (**Fig. 2E**). In lack of a saturating tendency, only lower bounds of *k*_2_’ could be determined, but the *K*_1_’*k*_2_’ value (the effective on-rate constant of ATP binding to acto-m5aS1) reflected in the slope of the plot was similar to that determined earlier (**Table 1**) [8], [17]. In acto-I67K-m5aS1, biphasic PA fluorescence transients were observed (**Fig. 2D**). The *k*_obs_ values of the rapid phase indicated slightly more rapid ATP binding kinetics compared to that of acto-wt-m5aS1 (**Fig. 2E**). The *k*_obs_ and amplitude profiles of the slow phase were similar to those observed for I67K-m5aS1 Trp transients (**Fig. 2E–F**; cf. **Fig. 2B–C**). We propose that the observed profiles of acto-I67K-m5aS1 reflect the formation of an off-pathway ATP binding intermediate (AM.ATP^#^ in **Fig. 1B**, see **Text S1**) with a kinetic behavior similar to that of M.ATP^#^, as determined from global fitting kinetic analysis (**Fig. S1C, Table S2**). The formation of this intermediate also resulted in a significant *y* intercept of the plot shown in **Fig. 2E**, despite the high overall affinity of ATP binding to acto-I67K-m5aS1 reflected in the amplitude profiles (**Fig. 2F**).

Light scattering profiles closely followed the inverse of the PA fluorescence transients in all cases, indicating that the detachment of m5aS1 from actin (*K*_8_ in **Fig. 1B**) is rapid and occurs practically simultaneously with the *K*_2_’ step.

**Figure 3:**
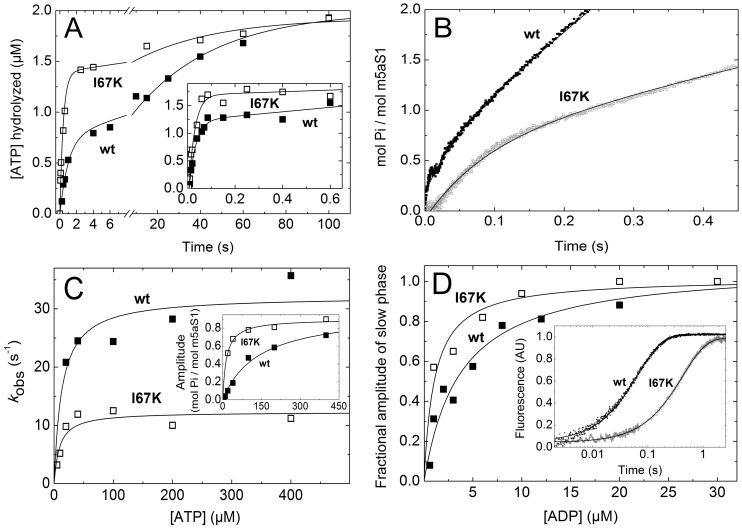
ATP hydrolysis and P_i_ release remain rapid and non-rate-limiting, but actin-activation of ADP release is abolished in I67K-m5aS1. ***A,*** Main panel: Single-turnover ATP hydrolysis profiles obtained on mixing 3.5 µM wt-m5aS1 (solid squares) or I67K-m5aS1 (open squares) with 2 µM γ-^32^P-ATP in the quenched-flow apparatus. Double exponential fits to datasets shown yielded *k*_obs_ values of 1.1 s^−1^ (37% fractional amplitude; limited by *K*_1_ *k*_2_) and 0.026 s^−1^ (limited by *k*_4_) for wt-m5aS1, and 2.2 s^−1^ (71% fractional amplitude) and 0.026 s^−1^ for I67K-m5aS1. Data for wt-m5aS1 were taken from [17]. Inset: Multiple-turnover ATP hydrolysis profiles obtained on mixing 3 µM wt-m5aS1 (solid squares) or I67K-m5aS1 (open squares) with 30 µM γ-^32^P-ATP in the quenched-flow apparatus. In the datasets shown, the rapid exponential burst had *k*_obs_ values (limited by *K*_1_ *k*_2_) of 35 s^−1^ and 39 s^−1^ with amplitudes of 1.2 µM (0.40 mol P_i_/mol m5aS1) and 1.7 µM (0.57 mol P_i_/mol m5aS1) for wt-m5aS1 and I67K-m5aS1, respectively. The slope of the linear steady-state phase (limited by *k*_4_) was 0.13 s^−1^ and 0.050 s^−1^ for wt-m5aS1 and I67K-m5aS1, respectively. *K*_3_ equilibrium constants were calculated from amplitude data as described in the text. ***B,*** Kinetic traces of P_i_ release (monitored by MDCC-PBP fluorescence) recorded on mixing 0.5 µM wt-m5aS1 (black trace) or I67K-m5aS1 (gray trace) plus 10 µM actin with 100 µM ATP in the stopped-flow apparatus. The wt-m5aS1 trace shown contained an exponential rapid burst with a *k*_obs_ of 24 s^−1^ and an amplitude of 0.44 mol P_i_/mol m5aS1, and a linear steady-state phase with a slope of 5.8 s^−1^. In the I67K-m5aS1 trace shown, the burst had a *k*_obs_ of 11 s^−1^ and an amplitude of 0.74 mol P_i_/mol m5aS1, and the steady-state slope was 1.7 s^−1^. ***C,*** ATP concentration dependence of *k*_obs_ (main panel) and amplitudes (inset) of the rapid burst in experiments performed as in ***B*** (solid squares, wt-m5aS1; open squares, I67K-m5aS1). Hyperbolic fits to *k*_obs_ datasets yielded maximal rate constants (*k*_max_ ≤ *k*_4_’) of 32 and 12 s^−1^, with half-saturation at 13 and 9.1 µM ATP for wt-m5aS1 and I67K-m5aS1, respectively. Hyperbolic fits to the amplitude datasets yielded maximal amplitudes of 0.99 and 0.90 mol P_i_/mol m5aS1 with half-saturation at 140 and 14 µM ATP for wt-m5aS1 and I67K-m5aS1, respectively. ***D,*** ADP release kinetics and ADP affinity of acto-m5aS1 monitored using PA fluorescence. Main panel, ADP concentration dependence of the fractional amplitudes of the slow phase of biphasic PA fluorescence transients recorded on rapidly mixing a premixture of 0.5 µM wt-m5aS1 (solid squares) or I67K-m5aS1 (open squares), 0.7 µM PA and the indicated ADP concentrations with 200 µM ATP in the stopped-flow apparatus (pre-mixing concentrations). In these conditions, the rapid and slow phases represented ATP-induced dissociation of the nucleotide-free and initially ADP-bound acto-m5aS1 fractions, respectively. In the case of the ADP-bound fraction, ADP release (*k*_5_’) limited the *k*_obs_ of acto-m5aS1 dissociation. Hyperbolic fits to the datasets yielded *K*_5_’ values of 3.7 and 1.0 µM for wt-m5aS1 and I67K-m5aS1, respectively. Inset: Representative transients obtained at a quasi-saturating ADP concentration (20 µM). The dominant slow phase had *k*_obs_ ( = *k*_5_’) values of 14 s^−1^ and 2.4 s^−1^ in wt-m5aS1 (black trace) and I67K-m5aS1 (gray trace), respectively.

We assessed the transient kinetic profile of the ATP hydrolysis step in the absence of actin (*K*_3_ in **Fig. 1B**) in quenched-flow experiments monitoring the cleavage of radioactively-labeled (γ-^32^P-) ATP upon mixing with m5aS1 in single-turnover and multiple-turnover conditions (**Fig. 3A**). In the biphasic profiles recorded in single-turnover experiments, the *k*_obs_ of the rapid phase was limited by the kinetics of ATP binding (*K*_1_ *k*_2_) at the applied ATP and protein concentrations (**Fig. 3A** main panel; cf. **Fig. 2A–C**, **Fig.** **S1** and **Table 1** **, Table S2**), whereas that of the slow phase was determined by the kinetics of P_i_ release (*k*_4_; see below). The equilibrium constant of the hydrolysis step (*K*_3_, calculated from the fractional amplitudes of the two phases as *A*_fast_/*A*_slow_) was increased by the I67K mutation (**Fig. 3A**, **Table 1**). Multiple-turnover kinetic profiles of ATP hydrolysis of wt-m5aS1 and I67K-m5aS1 consisted of a rapid exponential burst and a linear steady-state phase (**Fig. 3A** inset). The *k*_obs_ of the burst was still limited by ATP binding (*K*_1_ *k*_2_) at the applied ATP concentration (30 µM) (**Fig. 3A** inset; cf. **Fig. 2A–C**, **Fig.** **S1** and **Table 1** **, Table S2**), setting a lower bound for the effective rate constant of ATP hydrolysis (*k*_3_+ *k_–_*_3_, **Table 1**). *K*_3_ values calculated from burst amplitudes (*K*_3_ = *B*/(1-*B*) where *B* is the burst amplitude expressed in mol P_i_/mol m5aS1) were in line with those determined in single-turnover experiments (**Fig. 3A** inset and main panel, **Table 1**). The slope of the linear steady-state phase, representing the steady-state basal (actin-free) ATPase activity, appeared higher than that determined more robustly in P_i_ release and NADH-coupled steady-state experiments (**Fig. 3A** inset; see below). Taken together, quenched-flow experiments showed that I67K-m5aS1 retains rapid ATP hydrolysis, and the mutation facilitates the hydrolysis step (increases the *K*_3_ equilibrium constant; **Table 1**).

### I67K Mutant Retains Marked Activation of P_i_ Release by Actin

The kinetics of the release of P_i_ liberated from ATP by m5aS1 was monitored using a fluorescently-labeled P_i_ binding protein (MDCC-PBP) [22]. On mixing 0.5 µM wt-m5aS1 or I67K-m5aS1 with 100 µM ATP in the stopped-flow apparatus, no burst was observed before the linear steady-state phase of the reaction, indicating that P_i_ release (*k*_4_) is rate-limiting in both constructs in the absence of actin (**Table 1**). In the presence of 10 µM actin, the linear steady-state phase was preceded by an exponential burst whose *k*_obs_ and amplitude showed saturation with increasing ATP concentration (**Fig. 3B–C**). Maximal *k*_obs_ values were about three times lower in I67K-m5aS1 than in wt-m5aS1, whereas the maximal amplitudes were close to 1 mol P_i_/mol m5aS1 in both constructs (**Fig. 3B–C**, **Table 1**). The data indicate that, although actin-activated P_i_ release (*k*_4_’) is slowed down by the I67K mutation, ATP hydrolysis (*k*_3_+ *k*_–3_) and P_i_ release (*k*_4_’) remain rapid and non-rate-limiting in the presence of actin (**Table 1**).

### I67K Mutation Abolishes Activation of ADP Release by Actin

ADP release (*k*_5_’) from and ADP affinity (1/*K*_5_’) of acto-m5aS1 were monitored in PA fluorescence stopped-flow experiments via rapidly mixing a pre-mixture of PA-m5aS1 plus a range of ADP concentrations with excess ATP (**Fig. 3D**). In the biphasic PA fluorescence transients obtained in these conditions, the rapid phase represented ATP-induced dissociation of the ADP-free PA-m5aS1 fraction, whereas the slow phase originated from the PA-m5aS1 fraction that had initially bound ADP [17]. In the latter fraction, ADP release (*k*_5_’) limited the kinetics of the subsequent, ATP-induced dissociation of PA-m5aS1 (*K*_1_’*k*_2_’). The ADP release rate constants (*k*_5_’), reflected in the *k*_obs_ values of the slow phase of the transients (**Fig. 3D** inset), were compared to *k*_obs_ values of 3'-(N-methylanthraniloyl)-2'-deoxy-ADP (mdADP) fluorescence transients recorded on rapidly mixing m5aS1.mdADP complexes with excess ATP in the absence of actin (**Table 1**). The data indicated that the I67K mutation abolished the 7-fold activation of ADP release by actin, characteristic of wt-m5aS1 (**Table 1**). The ADP concentration dependence of the fractional amplitudes of the slow phase of PA fluorescence transients indicated that the high ADP affinity of acto-wt-m5aS1 (1/*K*_5_’) was further elevated by the I67K mutation (**Fig. 3D** main panel, **Table 1**).

### I67K Mutant Exhibits Slowed ATPase Activity and Retains High Steady-state Actin Attachment Ratio

**Figure 4:**
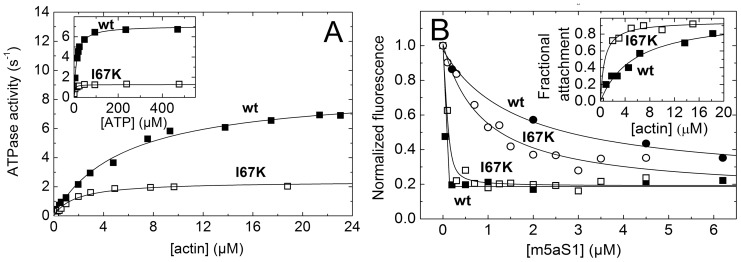
I67K mutant exhibits slowed actin-activated ATPase activity and high steady-state actin attachment. ***A,*** Actin (main panel) and ATP (inset) concentration dependence of wt-m5aS1 (solid squares) and I67K-m5aS1 (open squares) steady-state ATPase activity (concentrations used: 100 nM m5aS1 (both panels), 1 mM ATP (main panel), 10 µM actin (inset)). Hyperbolic fits to the datasets shown yielded maximal activities (*k*_cat_) of 8.7 and 2.4 s^−1^ with half-saturation (*K*_actin_) at 5.6 and 1.8 µM actin (main panel); and 7.1 and 1.3 s^−1^ with half-saturation (*K*_ATP_) at 12 and 2.8 µM ATP (inset) for wt-m5aS1 and I67K-m5aS1, respectively. ***B,*** Main panel, wt-m5aS1 (solid symbols) and I67K-m5aS1 (open symbols) concentration dependence of fluorescence intensities of 150 nM PA in the absence of nucleotide (rigor, squares) and in 1 mM ATP (circles). Quadratic fits to the rigor datasets (based on an equation described in [40]) and hyperbolic fits to the ATP datasets shown yielded apparent m5aS1 binding *K*_d_ values and m5aS1-saturated PA fluorescence levels (normalized to that in the absence of m5aS1) of less than 0.5 µM and 0.19 (for both wt-m5aS1 and I67K-m5aS1 in rigor); 1.7 µM and 0.23 (wt-m5aS1 in ATP); and 0.88 µM and 0.14 (I67K-m5aS1 in ATP). Inset, actin concentration dependence of the fractional actin attachment of 1 µM wt-m5aS1 (solid squares) or I67K-m5aS1 (open squares) in the presence of 15 mM ATP, determined in acto-m5aS1 cosedimentation experiments. Hyperbolic fits to the datasets indicated half-saturation at 5.5 and less than 2 µM actin for wt-m5aS1 and I67K-m5aS1, respectively.

As measured by an NADH-linked assay, the maximal actin-activated steady-state ATPase activity of wt-m5aS1 (*k*_cat_) was slowed down by about 6 times by the I67K mutation (**Fig. 4A**, **Table 1**), in parallel with the slowing of the rate constant of ADP release from acto-m5aS1 (*k*_5_’; **Fig. 3D** inset, **Table 1**). Ultracentrifugation-based acto-m5aS1 cosedimentation experiments performed in the presence of 15 mM ATP revealed that the high apparent actin affinity of wt-m5aS1 during steady-state ATPase cycling (originating from rate-limiting ADP release (*k*_5_’) and the high steady-state abundance of the acto-m5aS1.ADP ternary complex (**Fig. 1B**) [8], [17]), was further increased by the I67K mutation (**Fig. 4B** inset). Steady-state PA fluorescence intensities recorded upon titration with m5aS1 in the absence and presence of ATP indicated that strong actin binding by nucleotide-free m5aS1 was retained, and the apparent actin affinity during steady-state ATPase cycling was increased by the I67K mutation (**Fig. 4B** main panel). The data indicate that, similarly to wt-m5aS1, I67K-m5aS1 molecules spend a large proportion of their enzymatic cycle time in strongly actin-bound states (i.e. their duty ratio is high).

### I67K Mutation Interferes with Strong Actin Binding

We determined the kinetics of the binding of nucleotide-free m5aS1 to actin (*k*_–6_) by mixing m5aS1 (0.1–0.65 µM) with a 5-fold molar excess of PA in the stopped-flow apparatus. The PA concentration dependence of the *k*_obs_ of the transients revealed that the I67K mutation caused a 4-fold reduction in the rate constant of actin binding (*k*_–6_; **Table 1**). Chasing experiments performed by mixing PA-m5aS1 complexes with excess unlabeled actin revealed that the dissociation of nucleotide-free m5aS1 from actin (*k*_6_) was accelerated 7-fold by the mutation, resulting in a 28-fold reduction in the actin binding affinity (1/*K*_6_; **Table 1**).

### I67K Mutation Slows Down and Sensitizes Processive Motility

**Figure 5:**
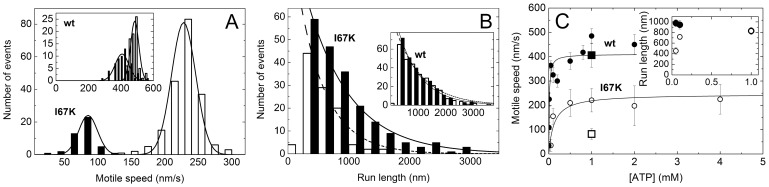
I67K mutation slows down and sensitizes processive motility. ***A,*** *In vitro* motile speeds of wt-m5aHMM (inset) and I67K-m5aHMM (main panel) at 1 mM ATP, measured in actin gliding (solid columns) and single-molecule TIRF (open columns) assays. Gaussian fits yielded average speeds shown in ***C***. ***B,*** Run lengths of wt-m5aHMM (inset) and I67K-m5aHMM (main panel) at 50 µM (open columns) and 1 mM (solid columns) ATP, determined in single-molecule TIRF experiments. Single exponential fits at 50 µM and 1 mM ATP (dashed and solid lines, respectively) yielded mean run lengths of 980±60 nm (wt-m5aS1, 50 µM ATP), 830±50 nm (wt-m5aS1, 1 mM ATP), 450±60 nm (I67K-m5aHMM, 50 µM ATP) and 830±70 nm (I67K-m5aHMM, 1 mM ATP) (see also panel ***C***). Data points below 250 nm were omitted from fits. ***C,*** ATP concentration dependence of average speeds (main panel) and mean run lengths (inset) of wt-m5aHMM (solid symbols) and I67K-m5aHMM (open symbols), measured in single-molecule TIRF (circles) and actin gliding (squares, main panel, 1 mM ATP) assays. Hyperbolic fits to the TIRF speed datasets (main panel) yielded maximal speeds (*v*_max_) and half-saturating ATP concentrations (*K*_ATP_) listed in **Table 1**. At 1 mM ATP, the run lengths (inset) of wt-m5aHMM and I67K-HMM were identical and the data points are indistinguishable. Error bars represent Gaussian half-widths (main panel) or standard errors of the fits (inset).

We assessed the effect of the I67K mutation on the motile properties of m5aHMM by recording the gliding of fluorescently-labeled actin filaments over m5aHMM-coated surfaces, and also by observing the movement of single GFP-labeled m5aHMM molecules on fixed actin filaments via TIRF (total internal reflection fluorescence) microscopy. In the applied conditions of the gliding assay (0.5–1 µM incubation concentration of m5aHMM), actin filaments were propelled by a multitude of m5aHMM molecules, possibly influencing each other’s actin translocating activity via mechanical linkage. In contrast, the TIRF measurement allowed the recording of the motility of individual m5aHMM molecules unaffected by other motors in the solution. In line with the reduced ADP release rate constant (*k*_5_’) and actin-activated ATPase activity (*k*_cat_) of I67K-m5aS1 (**Fig. 3D** inset, **4A**, **Table 1**), the motile speed of I67K-m5aHMM was significantly reduced compared to that of wt-m5aHMM, as measured by both types of motility assay (**Fig. 5A** **,** **Table 1**). Importantly, however, the actin gliding motile speed of I67K-m5aHMM was 3-fold reduced compared to the motile speed of single I67K-m5aHMM molecules, whereas only a slight reduction was observed in wt-m5aHMM (**Fig. 5A**). This finding indicates that the motility of mutant m5aHMM molecules was more strongly affected by mechanical drag exerted by multiple motor molecules bound to the same actin filament. The mean processive run length of single m5aHMM molecules was unaffected by the I67K mutation at 1 mM ATP (**Fig. 5B and C** inset). However, the run length of I67K-5aHMM was significantly reduced at lower ATP concentrations, unlike that of wt-m5aHMM (**Fig. 5B and C** inset). In addition, the motile speed of I67K-m5aHMM molecules reached half-saturation at a markedly higher ATP concentration (*K*_ATP_) than that of wt-m5aHMM (**Fig. 5C** **,** **Table 1**).

## Discussion

### Allosteric Effects of the Alteration of the NTS-converter Interface of m5a

The present study revealed that the alteration of the interface between the NTS and converter subdomains of m5a exerts marked and specific allosteric effects on the actin and ATP binding sites of the molecule. We found that the I67K mutation perturbed the strong-binding interaction of nucleotide-free m5a with actin, changed the mechanism of ATP binding, facilitated the ATP hydrolysis step, slightly slowed down actin-activated P_i_ release and abolished the actin-induced activation of ADP release (**Fig. 1B**, **Table 1**). The capability of processive mechanical stepping along actin was retained by mutant m5a, but its motility became slower and more sensitive to mechanical conditions (**Fig. 5**, **Table 1**). Below we discuss how these findings relate to and further our understanding of the structural and kinetic mechanism of actomyosin motility.

### Perturbation of the Strong-binding Actomyosin Interaction

Based on structural and kinetic considerations, the actin- and nucleotide-free (rigor-like) crystal structure of m5a (and those of molluscan muscle myosin 2 isoforms) are thought to closely resemble the myosin head conformation adopted in the strongly actin-bound (rigor) complex (**Fig. 1A**) [23]–[25]. This structure plays an important energetic role in the motile mechanism, as the formation of the strong-binding actomyosin interaction drives product release and the associated powerstroke [13].

The I67 and R710 side chains of chicken m5a (the latter being equivalent to R709 of mouse m5a) are in close juxtaposition in the rigor-like structure (**Fig. 1A**, **Table S1**). This arrangement suggests that the I67K mutation introduces significant charge repulsion between the side chains, similar to that observed for wt myosin 2 isoforms (**Fig. 1A**, **Table S1**). In line with this, we found that the strong-binding actin interaction (*K*_6_ in **Fig. 1B**) was significantly perturbed in I67K-m5aS1 (**Table 1**). Notably, in a crystallographically identified weakly ADP-bound m5a intermediate, the NTS-converter interface was similar to that in the rigor-like state (**Table S1**) [16]. Therefore the I67K mutation is likely to destabilize also this “weak-ADP” structure, providing a possible structural explanation for the mutation-induced abolishment of actin-activation of ADP release (see also below).

### Influence on ATP Binding Pathways

The binding of ATP to strongly actin-bound (rigor) m5aS1 weakens the actomyosin interaction by 5–6 orders of magnitude and leads to the formation of the actin-detached, strongly nucleotide-bound postrigor state (**Fig. 1A**; steps *K*_1_’, *K*_2_’ and *K*_8_ in **Fig. 1B**) [8], [9] [17]. We found that the I67K mutation left the main-flux ATP binding process largely unaffected, but induced the formation of a weakly ATP-bound off-pathway binding intermediate, which had similar kinetic properties in the absence and presence of actin (M.ATP^#^ and AM.ATP^#^ in **Fig. 1B** **;** **Fig. 2** **, Fig. S1, Table S2, Text S1**). These findings suggest that the NTS-converter interface exerts a subtle allosteric influence on the ATP binding pocket, and this influence is similar in actin-free (rigor-like) and actin-bound (rigor) m5a structures.

Our steady-state kinetic simulations based on the experimentally determined kinetic parameters of I67K-m5aS1 (**Table 1** **, Table S2**) indicated that, at saturating actin and ATP concentrations (20 µM and 5 mM, respectively), 19% of I67K-m5aS1 molecules populate the AM.ATP^#^ state during steady-state ATPase cycling, while M.ATP^#^ is insignificantly populated. The off-pathway ATP binding process exerts a slight (1.3-fold) slowing effect on the steady-state ATPase rate, but does not affect the high steady-state actin attachment of I67K-m5aS1 (close to 100%, cf. **Fig. 4B** inset).

Earlier we found that, in *Dd* myosin 2, the removal of the class-specific charge repulsion from the NTS-converter interface (K84-R704 interaction) did not affect the nucleotide binding process [15], in contrast to the effect of the introduction of this interaction into m5a in the present study (**Fig. 2A–F**). These findings are in line with structural data showing that the spatial separation of the affected interacting side chains occurs during the rigor-postrigor transition in m5a, whereas in myosin 2 the separation occurs later during the lever priming step (postrigor-prepowerstroke transition; **Fig. 1A**, **Table S1**).

The effect of the I67K m5a mutation on ATP binding is also different from those of previously-characterized m5a mutations located in the conserved switch-2 loop of the ATP binding pocket. The G440A mutation inhibited the ATP-induced weakening of the actomyosin interaction [9], [26], whereas mutations of the class-specific Y439 residue left the ATP binding process largely unaffected [17].

### Slowing of the Rate-limiting Step

During processive stepping along the actin filament, a double-headed m5a holoenzyme must maintain continuous attachment to actin, i.e. at least one head must be actin-bound at any time during the run. Therefore, a high duty ratio (a high fraction of the ATPase cycle time of a given head spent in strongly actin-bound states) is a prerequisite for processivity. In the ATPase cycle of wt m5a, the ATP-induced dissociation of the myosin head from actin (steps *K*_1_’, *K*_2_’ and *K*_8_ in **Fig. 1B**) is followed by rapid steps of actin-detached ATP hydrolysis (*K*_3_), rebinding to actin (*K*_9_) and actin-activated P_i_ release (*k*_4_’) [8], [17]. Thus, the subsequent release of ADP from actomyosin (*k*_5_’) will be rate-limiting in the overall enzymatic cycle. This kinetic pattern results in high steady-state abundance of the strongly-bound actomyosin.ADP complex, and thus it is the key kinetic determinant of the high duty ratio of wt m5a (≥70% for a single head) [8], [17]. The release of ADP leads to the transient formation of the nucleotide-free actomyosin rigor complex. The myosin head then rapidly binds a new ATP molecule and dissociates from actin. The results of the present study showed that ADP release (*k*_5_’) and actin-activated steady-state ATPase activity (*k*_cat_) were slowed down to a similar extent by the I67K mutation (6–7 times), indicating that ADP release remained rate-limiting also in the mutant enzyme (**Fig. 3D** **,** **4A** **,** **Table 1**). Accordingly, the high duty ratio of wt-m5aS1 was retained (or even elevated) in the mutant construct (**Fig. 4B**). The slowing of ADP release resulted from the mutation-induced abolishment of the actin-induced kinetic activation of this step (**Table 1**).

It was shown earlier that the kinetics of actin-activated ADP release is limited by a structural transition of the nucleotide binding pocket occurring in the actomyosin.ADP state, prior to ADP release [27]. The mechanical load dependence of this step could ensure that a m5a head generating force is inhibited from releasing ADP until the working stroke is complete. This transition may well be affected by the I67K mutation, similar to earlier characterized mutations of the class-specific Y439 residue of the switch-2 loop of m5a [17]. The observation that the mutations abolished the actin-induced activation of ADP release is consistent with this idea.

### Sensitization of Processive Motility

While I67K-m5aHMM retained the capability of processive motion along actin filaments (**Fig. 5B–C**), the abolishment of the actin-activation of ADP release by the mutation (**Fig. 3D**) resulted in significant slowing of motile speeds (**Fig. 5A,C**). Importantly, the slowing effect was markedly stronger in the ensemble motility arrangement (when actin filaments were propelled by multiple m5aHMM molecules exerting a mechanical drag on each other) than that observed for the independent motion of single m5aHMM molecules along actin (**Fig. 5A,C** **,** **Table 1**). These findings imply that the unloaded motile speed was slowed and the force sensitivity was enhanced by the mutation. However, it should be noted that other factors may also influence the observed phenomena. The higher overall actin affinity of I67K-m5a during steady-state ATPase cycling (**Fig. 4B**) is likely to pose an additional drag on actin filaments, which may reduce the motile speed in the ensemble assay, as demonstrated in frictional loading assays using actin-binding proteins [28]–[30]. In addition, being located at the base of the lever, the I67K mutation may influence the stiffness of the head, leading to a lower isometric force. Interestingly, the observed difference between ensemble and single-molecule behavior was absent in the previously-characterized Y439A (switch-2) mutant of m5a, despite the similarity of Y439A and I67K mutations in abolishing the actin-induced activation of ADP release (**Fig. 3D**) [**17**].

In summary, our data revealed that the perturbation of the NTS-converter interface of m5a allosterically affects ATP binding, hydrolysis and product release, resulting in slower and mechanically sensitized processive motility. These results highlight that the efficient functioning of myosins requires fine-tuned allosteric linkages shaped by widely differing physiological demands.

## Materials and Methods

### Proteins

Cloning, expression and purification of wt-m5aS1 and wt-m5aHMM (with heavy chains comprising the first 820 and 1091 amino acid residues of m5a, respectively) were described in [17] and [31], [32], respectively. I67K point mutant m5aS1 and GFP-m5aHMM clones were constructed via QuikChange (Stratagene) using a complementary oligonucleotide pair with a coding-strand sequence of CCTCACTTACGGAACCCTGACAAGCTTGTTGGAGAAAATGACC (mutant triplet underlined). All DNA constructs were verified by DNA sequencing.

Actin was purified as in [33] and pyrene-labeled as in [34].

### Solution Kinetic and Spectroscopic Experiments

All experiments were performed at 25°C in a buffer comprising 20 mM HEPES (pH 7.0), 50 mM KCl, 5 mM MgCl_2_, 0.1 mM EGTA, and 1 mM DTT. In transient kinetic experiments the volume mixing ratio was 1∶1 and post-mix concentrations are stated unless otherwise indicated. In the experiments of **Fig. 3B–C**, MDCC-PBP fluorescence values were converted to P_i_ concentration based on calibration described in [35]. Cosedimentation assays (**Fig. 4B** inset) were carried out as in [36]. Steady-state Trp fluorescence emission spectra were recorded as in [36]. Other details of sample preparation, assay components, optical setups and data analysis were described in [17]. Global fitting kinetic analysis and steady-state kinetic simulations were performed using KinTek Explorer 3.0 software [37].

### *In vitro* Motility and TIRF Microscopic Experiments

*In vitro* actin gliding and TIRF motility assays were performed as described in [17], [38], [39]. All experiments were performed at 25°C controlled by an enclose box in an OLYMPUS IX70 microscope (Olympus, Tokyo, Japan) [17], [31] in a buffer used in [17].

## Supporting Information

Figure S1 **Global fitting analysis of I67K-m5aS1 and acto-I67K-m5aS1 ATP binding transients.** Dots represent normalized transients of (***A***) Trp fluorescence recorded upon mixing 0.5 µM I67K-m5aS1 with ATP (cf. **Fig. 2A**), (***B***) mdATP fluorescence recorded upon mixing 0.5 µM I67K-m5aS1 with mdATP, and (***C***) PA fluorescence recorded upon mixing 0.35 µM PA plus 0.25 µM I67K-m5aS1 with ATP (cf. **Fig. 2D**) in the stopped-flow apparatus. Nucleotide concentrations (in µM) are indicated in panel legends. Global fitting analysis was performed based on a model consisting of the steps denoted in **Fig. 1B** as *K*_1_, *K*_2_, *K*_3_ and *K*_#_ (***A***); *K*_1_, *K*_2_, and *K*_#_ (***B***); or *K*_1_’, *K*_2_’, *K*_8_ and *K*_#_’ (***C***). As mentioned in Results, the *K*_1_ and *K*_2_ (or *K*_1_’ and *K*_2_’) steps could not be separately resolved in these experiments. Thus, these steps were merged into a single binding step in which *k*_on_ (***A–B***) or *k*_on_’ (***C***) were equivalent to *K*_1_ *k*_2_ or *K*_1_’*k*_2_’, respectively. The signal change was modeled to occur on the *K*_3_ (***A***), *K*_2_ (***B***) or *K*_2_’ (***C***) steps. All other steps, including *K*_#_ and *K*_#_’, were modeled as optically silent. In ***A***, the apparent rate constant of the *K*_3_ step (*k*_3_+ *k*_–3_) could be robustly determined, whereas their ratio (*K*_3_, determined robustly in the quenched-flow experiments of **Fig. 3A**) did not influence the Trp fluorescence transient profiles. In ***C***, *K*_8_ was modeled as a rapid and irreversible step, as inferred from the match between PA fluorescence and light scattering transient profiles (see Results). Variable *y* offset and total amplitude correction factors were used in the global fits to obtain the best-fit model. Lines represent simulations based on best-fit parameters determined by global fitting kinetic analysis. For the datasets shown, best-fit parameters were the following: (***A***) *k*_on_ = 1.69 µM^−1^s^−1^, *k*_3_+ *k*_–3_ = 635 s^−1^, *k*_#_ = 0.117 µM^−1^s^−1^, *k*_–#_ = 3.77 s^−1^; (***B***) *k*_on_ = 2.17 µM^−1^s^−1^, *k*_#_ = 0.179 µM^−1^s^−1^, *k*_–#_ = 5.05 s^−1^; (***C***) *k*_on_’ = 0.511 µM^−1^s^−1^, *k*_#_’ = 0.219 µM^−1^s^−1^, *k*_–#_’ = 2.17 s^−1^. Best-fit *k*_–2_ (***A–B***) or *k*_–2′_ (***C***) values, representing the effective on-pathway ATP dissociation rate constant, were not significantly different from zero in any experiment, indicating that the productive (on-pathway) ATP binding process (*K*_1_ *k*_2_ in ***A–B*** or *K*_1_’*k*_2_’ in ***C***) is quasi-irreversible. **Table S2** shows the statistics of best-fit parameters obtained in all I67K-m5aS1 and acto-I67K-m5aS1 ATP binding experiments. The robustness of global fits is indicated by the fact that the means and SD values for the best-fit parameters were similar to those of corresponding *k*_on_ parameters resulting from exponential fitting analysis (**Fig. 2**, **Table 1**). Global fitting analysis yielded parameters identical to those of the exponential fitting analysis (**Fig. 2**, **Table 1**) also in the case of wt-m5aS1 and acto-wt-m5aS1.
(TIF)

Table S1 **Distances characteristic of the NTS-converter interaction (between residues homologous to I67 and R710 of chicken m5a) in different structural states of various myosin isoforms.** ^a^ *Gg, Gallus gallus*; MDE, MD plus essential light chain construct; *Dd, Dictyostelium discoideum*; *Lp, Loligo pealei; Pm, Placopecten magellanicus*; *Ai, Argopecten irradians.*
(DOCX)

Table S2 **Results of global fitting analysis of I67K-m5aS1 and acto-I67K-m5aS1 nucleotide binding transients^ a^**. ^a^Nomenclature of kinetic constants refers to **Fig. 1B**. Mean ± SD values of best-fit parameters for two independent sets of experiments are shown. See **Fig. S1** for details of modeling and simulation, and **Table 1** for corresponding *k*_on_ and *k*_on_’ parameters resulting from exponential fitting analysis. ^b^ Global fitting and exponential analysis of monophasic mdATP binding transients of wt-m5aS1 yielded *k*_on_ ( = *K*_1_ *k*_2_) = 1.6±0.3 µM^−1^s^−1^.
(DOCX)

Text S1 **Interpretation of I67K-m5aS1 and acto-I67K-m5aS1 ATP binding transients.**
(DOCX)
