# Supplementary figures and images for: A Subdomain Interaction at the Base of the Lever Allosterically Tunes the Mechanochemical Mechanism of Myosin 5a
Source: PLoS One. 2013 May 1;8(5):e62640. doi: 10.1371/journal.pone.0062640 (PMC3641075; doi:10.1371/journal.pone.0062640)

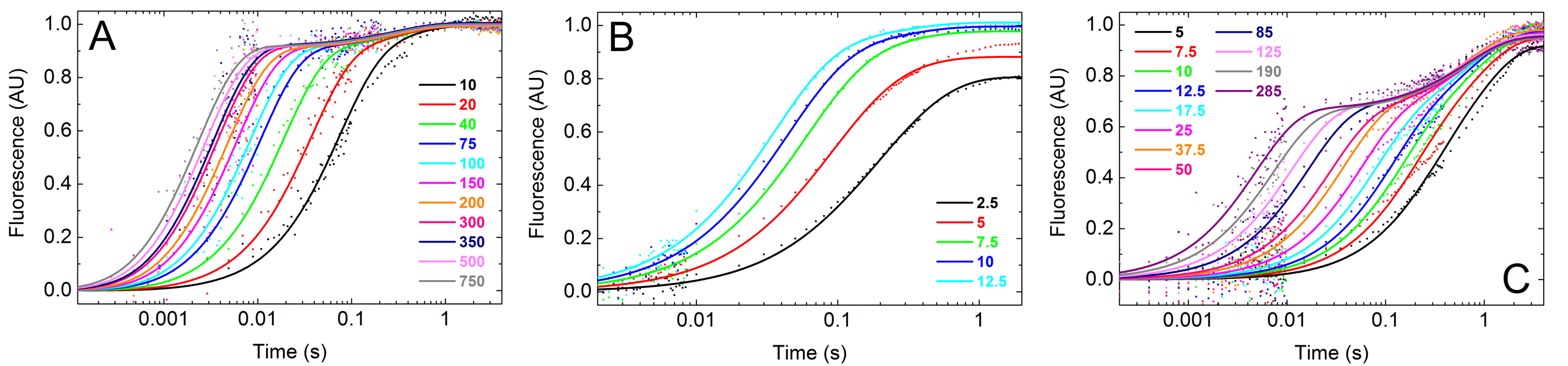

Supplement: Figure S1 — Global fitting analysis of I67K-m5aS1 and acto-I67K-m5aS1 ATP binding transients. Dots represent normalized transients of (A) Trp fluorescence recorded upon mixing 0.5 µM I67K-m5aS1 with ATP (cf. Fig. 2A ), (B) mdATP fluorescence recorded upon mixing 0.5 µM I67K-m5aS1 with mdATP, and (C) PA fluorescence recorded upon mixing 0.35 µM PA plus 0.25 µM I67K-m5aS1 with ATP (cf. Fig. 2D ) in the stopped-flow apparatus. Nucleotide concentrations (in µM) are indicated in panel legends. Global fitting analysis was performed based on a model consisting of the steps denoted in Fig. 1B as K 1, K 2, K 3 and K # (A); K 1, K 2, and K # (B); or K 1’, K 2’, K 8 and K #’ (C). As mentioned in Results, the K 1 and K 2 (or K 1’ and K 2’) steps could not be separately resolved in these experiments. Thus, these steps were merged into a single binding step in which k on (A–B) or k on’ (C) were equivalent to K 1 k 2 or K 1’k 2’, respectively. The signal change was modeled to occur on the K 3 (A), K 2 (B) or K 2’ (C) steps. All other steps, including K # and K #’, were modeled as optically silent. In A, the apparent rate constant of the K 3 step (k 3+ k –3) could be robustly determined, whereas their ratio (K 3, determined robustly in the quenched-flow experiments of Fig. 3A ) did not influence the Trp fluorescence transient profiles. In C, K 8 was modeled as a rapid and irreversible step, as inferred from the match between PA fluorescence and light scattering transient profiles (see Results). Variable y offset and total amplitude correction factors were used in the global fits to obtain the best-fit model. Lines represent simulations based on best-fit parameters determined by global fitting kinetic analysis. For the datasets shown, best-fit parameters were the following: (A) k on = 1.69 µM−1s−1, k 3+ k –3 = 635 s−1, k # = 0.117 µM−1s−1, k –# = 3.77 s−1; (B) k on = 2.17 µM−1s−1, k # = 0.179 µM−1s−1, k –# = 5.05 s−1; (C) k on’ = 0.511 µM−1s−1, k #’ = 0.219 µM−1s−1, k –#’ = 2.17 s−1. Best-fit k [file pone.0062640.s001.tif]
