# Supplementary material for: A Subdomain Interaction at the Base of the Lever Allosterically Tunes the Mechanochemical Mechanism of Myosin 5a
Source: PLoS One. 2013 May 1;8(5):e62640. doi: 10.1371/journal.pone.0062640 (PMC3641075; doi:10.1371/journal.pone.0062640)
Supplement: Table S1 — Distances characteristic of the NTS-converter interaction (between residues homologous to I67 and R710 of chicken m5a) in different structural states of various myosin isoforms. a Gg, Gallus gallus; MDE, MD plus essential light chain construct; Dd, Dictyostelium discoideum; Lp, Loligo pealei; Pm, Placopecten magellanicus; Ai, Argopecten irradians. (DOCX) [file pone.0062640.s002.docx]

| **PDB code** | **Construct ^a^** | **Nucleotide ligand** | **Conformation** | **Residue pair** | **Distance (Å)** | **Atoms used in distance calculation** |
| --- | --- | --- | --- | --- | --- | --- |
| 1OE9 | *Gg* m5a MDE | none | near-rigor | I67-R710 | 3.4 | C_δ_-C_ε_ |
| 1W7I |  | ADP | weak-ADP |  | 3.7 | C_δ_-C_ε_ |
| 1W7J |  | ADP.BeF_x_ | postrigor |  | 15.6 | C_δ_-C_ε_ |
| 1MMD | *Dd* myosin *2* MD | none | near-rigor | K84-R704 | 4.5 | N_ζ_-C_ε_ |
| 1Q5G |  | ADP.BeF_x_ | postrigor |  | 4.9 | N_ζ_-C_ε_ |
| 1VOM |  | ADP.V_i_ | prepowerstroke |  | 35.5 | N_ζ_-C_α_ |
| 3I5G | *Lp* myosin *2* S1 | none | near-rigor | K81-R721 | 6.6 | N_ζ_-C_ε_ |
| 3I5F |  | ADP | postrigor |  | 4.6 | N_ζ_-C_ε_ |
| 2OS8 | *Pm* catch myosin 2 S1 ^b^ | none | near-rigor | K81-R719 | 5.1 | N_ζ_-C_ε_ |
| 2OTG |  | ADP | postrigor |  | 4.1 | N_ζ_-C_ε_ |
| 1SR6 | *Ai* myosin 2 S1 | none | postrigor | K81-R719 | 3.9 | N_ζ_-C_ε_ |
| 1QVI |  | ADP.V_i_ | prepowerstroke |  | 27.3 | N_ζ_-C_ε_ |
