# Supplementary material for: A Subdomain Interaction at the Base of the Lever Allosterically Tunes the Mechanochemical Mechanism of Myosin 5a
Source: PLoS One. 2013 May 1;8(5):e62640. doi: 10.1371/journal.pone.0062640 (PMC3641075; doi:10.1371/journal.pone.0062640)
Supplement: Table S2 — Results of global fitting analysis of I67K-m5aS1 and acto-I67K-m5aS1 nucleotide binding transients a. aNomenclature of kinetic constants refers to Fig. 1B . Mean ± SD values of best-fit parameters for two independent sets of experiments are shown. See Fig. S1 for details of modeling and simulation, and Table 1 for corresponding k on and k on’ parameters resulting from exponential fitting analysis. b Global fitting and exponential analysis of monophasic mdATP binding transients of wt-m5aS1 yielded k on ( = K 1 k 2) = 1.6±0.3 µM−1s−1. (DOCX) [file pone.0062640.s003.docx]

| **Parameter** | **Method of determination** | **Value** |
| --- | --- | --- |
| **ATP binding to m5aS1** | Trp fluorescence |  |
| *k*_on_ (= *K*_1_*k*_2_) (µM^-1^s^-1^) |  | 0.98 ± 0.33 |
| *k*_3_ + *k*_–3_ (s^-1^) |  | 810 ± 110 |
| *k*_#_ (µM^-1^s^-1^) |  | 0.17 ± 0.02 |
| *k_–_*_#_ (s^-1^) |  | 6.7 ± 2.0 |
| **mdATP binding to m5aS1** | mdATP fluorescence |  |
| *k*_on_ (= *K*_1_*k*_2_) (µM^-1^s^-1^) ^b^ |  | 1.7 ± 0.5 |
| *k*_#_ (µM^-1^s^-1^) |  | 0.11 ± 0.07 |
| *k_–_*_#_ (s^-1^) |  | 3.9 ± 1.1 |
| **ATP binding to acto-m5aS1** | PA fluorescence, light scattering |  |
| *k*_on_’ (= *K*_1_’*k*_2_’) (µM^-1^s^-1^) |  | 1.2 ± 0.5 |
| *k*_#_’ (µM^-1^s^-1^) |  | 0.32 ± 0.10 |
| *k_–_*_#_’ (s^-1^) |  | 2.3 ± 0.2 |
