# Supplementary material for: A Subdomain Interaction at the Base of the Lever Allosterically Tunes the Mechanochemical Mechanism of Myosin 5a
Source: PLoS One. 2013 May 1;8(5):e62640. doi: 10.1371/journal.pone.0062640 (PMC3641075; doi:10.1371/journal.pone.0062640)
Supplement: Text S1 — Interpretation of I67K-m5aS1 and acto-I67K-m5aS1 ATP binding transients. (DOCX) [file pone.0062640.s004.docx]

**Interpretation of I67K-m5aS1 and acto-I67K-m5aS1 ATP binding transients**

In principle, the ATP concentration dependence of the biphasic I67K-m5aS1 Trp fluorescence (**Fig. 2A-C**) and acto-I67K-m5aS1 PA fluorescence (**Fig. 2D-F**) transient profiles could be interpreted in the framework of a multistep ATP interaction mechanism without an off-pathway binding intermediate. In such a mechanism, the initial binding process (reflected in the rapid phase of the transients) would be followed by a slow step occurring in the main pathway of the ATPase reaction, giving rise to the slow phase of the transients. However, we excluded this possibility based on the following considerations.

(*i*) In the absence of actin, the maximal *k*_obs_ of the slow phase of Trp fluorescence transients was in the range of 6-8 s^-1^ (**Fig. 2B** inset). However, multiple-turnover quenched-flow results (**Fig. 3A** inset) showed that ATP hydrolysis (*k*_3_ + *k*_–3_) occurs at least at a rate constant of 39 s^-1^, excluding that the slow step precedes or is equivalent to ATP hydrolysis (*K*_3_). In the same experiments (**Fig. 3A** inset), the lack of a second exponential phase excludes the possibility of a slow (6-8 s^-1^) step following reversible ATP hydrolysis (*k*_3_ + *k*­_–3_ > 39 s^-1^) but preceding the rate-limiting P_i_ release (*k*_4_ = 0.025 s^-1^; **Table 1**).

(*ii*) In the presence of actin, the maximal *k*_obs_ of the slow phase of PA fluorescence transients was in the range of 3-4 s^-1^ (**Fig. 2E** inset). However, MDCC-PBP fluorescence transients recorded upon mixing acto-I67K-m5aS1 with excess ATP revealed that P_i_ release (*k*_4_’) occurs at least at a rate constant of 12 s^-1^ (**Fig. 3C**, **Table 1**), excluding the possibility of any slower on-pathway process during ATP binding (*K*_1_’*k*_2_’), acto-m5aS1 dissociation (*K*_8_) and ATP hydrolysis (*K*_3_).

It has been proposed for several myosin isoforms that nucleotide-free acto-S1 can reversibly adopt two conformations, with one conformation being incapable of nucleotide binding [1-4]. The ATP binding kinetics of these isoforms is thus influenced by the conformational equilibrium of nucleotide-free acto-S1. However, this scenario can be excluded in the case of both I67K-m5aS1 and acto-I67K-m5aS1, based on the inverse ATP concentration dependence of rapid- and slow-phase amplitudes of the ATP binding transients (**Fig. 2C,F**).

Based on the above considerations, we propose that the kinetic behavior of I67K-m5aS1 and acto-I67K-m5aS1 (**Fig. 2**) reflects the reversible formation of off-pathway ATP-bound intermediates (M.ATP^#^ and AM.ATP^#^, respectively; **Fig. 1B**). We verified this model by global fitting analysis (see Results; **Fig. S1**, **Table S2**).

**REFERENCES**

1. Yang Y, Kovacs M, Sakamoto T, Zhang F, Kiehart DP, et al. (2006) Dimerized Drosophila myosin VIIa: a processive motor. Proc Natl Acad Sci U S A 103: 5746-5751.

2. Yang Y, Kovacs M, Xu Q, Anderson JB, Sellers JR (2005) Myosin VIIB from Drosophila is a high duty ratio motor. J Biol Chem 280: 32061-32068.

3. Geeves MA, Perreault-Micale C, Coluccio LM (2000) Kinetic analyses of a truncated mammalian myosin I suggest a novel isomerization event preceding nucleotide binding. J Biol Chem 275: 21624-21630.

4. Jontes JD, Milligan RA, Pollard TD, Ostap EM (1997) Kinetic characterization of brush border myosin-I ATPase. Proc Natl Acad Sci U S A 94: 14332-14337.
